# Reverse Total Shoulder Arthroplasty in the Setting of an Intact Rotator Cuff: A Contemporary Review of Current Indications, Outcomes, and Surgical Considerations

**DOI:** 10.1007/s12178-026-10066-3

**Published:** 2026-10-26

**Authors:** Liam H. Wong, Benjamin M. Lurie, Michael R. Davies

**Affiliations:** Department of Orthopaedic Surgery, University of California, San Fracisco, 1500 Owens St, San Francisco, CA 94158 USA

**Keywords:** Reverse total shoulder arthroplasty, intact rotator cuff, glenohumeral osteoarthritis, shoulder arthroplasty, reverse total shoulder arthroplasty outcomes

## Abstract

**Purpose of Review:**

Reverse total shoulder arthroplasty (rTSA) was designed for rotator cuff tear arthropathy but is now used in patients with an intact rotator cuff, most often for primary glenohumeral osteoarthritis (GHOA). This review examines the current evidence for rTSA in cuff-intact patients, focusing on the indications, functional outcomes, complications, implant design considerations and surgical technique.

**Recent Findings:**

In matched cohorts and meta-analyses restricted to cuff-intact GHOA, rTSA and aTSA produce comparable pain relief, patient-reported outcomes, satisfaction, and overhead elevation. ATSA restores greater external and internal rotation, but this difference often falls below clinically important thresholds and narrows over time. RTSA generally has a lower overall complication and revision rate, with baseplate loosening as the leading though still rare reason for revision surgery. Instability, acromial and scapular spine stress fracture, and revision surgery rates are all lower when the rotator cuff is intact. Current studies on the cuff-intact population are limited to 10-year outcomes, with registry survivorship of rTSA for GHOA approximately 96.1% at 10 years.

**Summary:**

RTSA is an efficacious and durable option for patients with glenohumeral arthritis and an intact rotator cuff, providing consistent pain relief and function. Patient outcomes are influenced by surgical technique, implant design and positioning, and avoidance of complications. Long-term follow-up and further comparative studies are needed to define the role of rTSA versus aTSA in this population.

## Introduction

The utilization of reverse total shoulder arthroplasty (rTSA) has grown significantly over the past two decades. Registry analyses report a more than twofold increase in U.S. rTSA volume between 2011 and 2021 [1, 2]. Much of this reflects expansion beyond the procedure’s original indication of rotator cuff tear arthropathy into a broader range of diagnoses, including primary glenohumeral osteoarthritis (GHOA) in patients with an intact rotator cuff [3, 4]. This expansion has been driven by increased surgeon familiarity with the procedure, the reliable long-term fixation and in-growth in patients with glenoid bone-loss and/or deformity, and the ability of the prosthesis to function irrespective of progressive degeneration of the rotator cuff after surgery [5,6]. However, the use of rTSA in shoulders with an intact rotator cuff represents a technically off-label application, as the original 2003 FDA clearance was for arthritis in the setting of cuff tear arthropathy or a grossly rotator cuff-deficient joint, which remains the only FDA-approved indication [7].

Although anatomic total shoulder arthroplasty (aTSA) has been the preferred treatment for primary GHOA, current evidence supports rTSA as a legitimate primary option for GHOA with an intact rotator cuff. In matched cohorts and meta-analyses restricted to cuff-intact GHOA, rTSA and aTSA deliver comparable pain relief, patient-reported outcomes (PROs), satisfaction, and overhead forward elevation (FE). ATSA retains an advantage in external (ER) and internal rotation (IR), though this is frequently below clinically important thresholds and may narrow over longer-term follow-up [6, 8-10]. With appropriate patient selection, implant positioning and surgical technique, rTSA is a durable and reliable construct. Its complication burden and long-term implant survivorship remain under study, with ongoing concerns about the high morbidity associated with rare complications such as baseplate failure, stress fracture, dislocation and infection [8, 11-13]. Long-term survivorship data in this population are currently limited to series with less than 10 years of follow-up.

The purpose of this review was to synthesize the current evidence for rTSA in patients with an intact rotator cuff, discussing appropriate indications, functional outcomes, comparison with aTSA, complication profiles, and the role of implant design. We offer perspectives on patient selection and technical considerations informed by the emerging comparative literature.

## Indications for Reverse Total Should Arthroplasty in the Intact Rotator Cuff Patient

### Primary Glenohumeral Osteoarthritis

Since its development, aTSA has been the preferred treatment for primary GHOA, offering reliable pain control, improvements in both FE and rotational range of motion (ROM), and excellent intermediate-term outcomes [14]. However, despite improvements in implant design and surgical technique, complications such as subscapularis failure, glenoid polyethylene loosening and osteolysis, instability, posterior-superior cuff failure, and stiffness remain prevalent, particularly at long-term follow-up. RTSA is being explored for patients with primary GHOA who carry patient-specific or anatomic risk factors for complications or poor outcomes after aTSA [4]. Older age, significant medical comorbidities, greater glenoid bone loss or deformity, prior shoulder surgery, and markedly limited preoperative ROM all increase risk for complications and worse functional outcomes after aTSA [15].

In the geriatric population, many studies have shown equivalent or superior outcomes with rTSA. Patients ≥ 70 years had lower complication (Relative Risk [RR] 1.75) and revision rates (RR 3.96) and equivalent PROs when treated with rTSA [6, 14]. Ardebol et al. found that patients ≥ 75 years with primary GHOA and an intact rotator cuff achieved similar postoperative and overall improvement to PROs (overall satisfaction, American Shoulder and Elbow Surgeons [ASES], Subjective Shoulder Value [SSV], Visual Analog Scale [VAS]) and ROM (FF, IR) after either rTSA or aTSA [16], and a systematic review of rTSA performed specifically for cuff-intact GHOA confirmed reliable pain relief and functional gains across the series [17].

Patients with limited preoperative FE (often defined as < 90°) represent a subgroup that may have a functionally, if not structurally, impaired rotator cuff and increased risk of worse outcomes after aTSA. Trammel et al. matched 344 aTSA patients to 163 rTSA patients (all with ≤ 105° of passive FE) and found that rTSAs had greater improvements in active FE and abduction, superior PROs, and lower revision surgery rates compared to aTSAs, though the overall complication rate did not differ [18]. However, Wright et al. found that patients ≥ 70 years with active FE below 90° had similar postoperative ROM, PROs, and complication rates [19]. Similarly, Karimi et al. found that although rTSA patients had greater FE at three months postoperatively (129° vs. 102°), there was no difference in PROs, FE at 6 months, or overall complication rates, and rTSA patients had less IR compared to those who underwent aTSA [18, 20].

Glenoid retroversion, posterior humeral subluxation, and glenoid bone loss increase the risk of glenoid loosening and recurrent posterior humeral head subluxation after aTSA [21]. Patients with Walch B2 and B3 glenoid morphology may have lower complication rates and increased implant survival when treated with rTSA [9, 22]. The posterior-inferior bone loss typical of advanced primary GHOA alters the joint center-of-rotation, produces static posterior subluxation, and changes soft-tissue tension [5]. The eccentric posterior loading is thought to drive early glenoid polyethylene loosening via the rocking horse phenomenon, a principal long-term complication of aTSA with B2 and B3 glenoid morphologies [21, 23, 24]. RTSA, which generally achieves initial baseplate stability with multiple screws and long-term stability through bony ingrowth, may lead to lower rates of component loosening and improve revision-free implant survival [25].While numerous techniques have been described to mitigate the challenges of performing aTSA with B2 and B3 glenoid morphologies (eccentric reaming, augmented glenoid polyethylene components, inset or inlay glenoid components), no technique has completely eliminated polyethyelene loosening, instability, and recurrent posterior subluxation [5, 9, 26]. It is important to note that while radiolucency around a glenoid baseplate and polyethylene are common with rates up to 16% in rTSA and 41 − 51% in aTSA with over 10 years median follow-up, this often does not correlate with PROs or the need for revision surgery, with revision rates of 2% for rTSA and 17% for aTSA [27–29]. In a propensity-matched cohort of 101 aTSA versus 101 rTSA patients with B2 or B3 wear and an intact cuff, Polisetty et al. reported comparable short-term outcomes with rTSA achieving a marginally lower postoperative pain score (0.5 vs. 1.2; *P* = 0.036) [22]. However, they found 29 aTSA cases had glenoid radiolucencies (3 grossly loose) versus only two complete baseplate loosening cases in rTSA (1 following trauma) (*P* < 0.001) [22]. A 2025 systematic review of 36 studies (1349 aTSA and 478 rTSA patients with cuff-intact primary GHOA) found comparable improvements in ROM, ASES, Constant, and VAS scores for B2 and B3 glenoids [30].

The long-term durability of PRO improvements after aTSA versus rTSA remains unknown, with multiple factors potentially influencing long term outcomes including implant stability, progressive rotator cuff pathology, and deltoid fatigue. In a retrospective comparison of 194 patients with primary GHOA and eccentric glenoid wear, Cuff et al. found that aTSA with high-side reaming yielded superior early functional scores and ROM at 2 years including better FE (162° vs. 151°, *P* < 0.001), ER (47° vs. 42°, *P* < 0.001), and IR (80% vs. 55% full IR, *P* < 0.001), but these advantages diminished by minimum 7-year follow-up. At final follow-up, FE had converged (149° vs. 152°, *P* = 0.025), while aTSA retained only modest advantages in ER (45° vs. 41°, *P* < 0.001) and IR (74% vs. 54% full IR, *P* = 0.005). Meanwhile, rTSA demonstrated higher ASES scores (80 vs. 77, *P* < 0.001), higher patient satisfaction (95% vs. 82%), and a lower revision rate (2% vs. 8%) at 7 years. Similarly, Leinweber et al. found in a propensity-matched comparison of 122 patients with primary GHOA and varying degrees of rotator cuff pathology that early aTSA advantages in ER (63.0° vs. 57.0°, *P* = 0.036) and IR (5.0 vs. 3.3, *P* < 0.001) at 1 − 2 years were no longer statistically significant at midterm 4 − 6 year follow-up (ER 63.7° vs. 60.0°; IR 4.1 vs. 4.5; *P* > 0.21), and that early differences in substantial clinical benefit also diminished over time [10]. This appears to be due to late degradation of PROs in aTSA rather than further improvement in rTSA outcomes.

Secondary rotator cuff failure remains the second most common reason for revision of aTSA. Young et al. found that while 84% of aTSA patients remained free of symptomatic cuff dysfunction at 10 years, this dropped to 45% at 15 years [31]. Longer-term studies have reported even higher rates of cuff-related pathology. Lafosse et al. found 44% superior humeral head migration at a mean 12.4 years [32], and Ranieri et al. reported 44% cuff deficiency in a systematic review of 1,163 all-polyethylene glenoid aTSAs at a mean 11.8 years, though the overall revision rate was only 17% [28]. Importantly, the accuracy of the humeral reconstruction and re-creation of native center-of-rotation in aTSA is not accounted for in most large, long-term studies. Rotator cuff failure, whether acute or attritional, is not an indication for rTSA revision.

### Inflammatory Arthritis

Inflammatory arthritis is a commonly described indication for rTSA. Long-standing rheumatoid and other inflammatory arthritis produces glenoid and humeral bone erosion, poor bone quality, and rotator cuff attenuation. RTSA is frequently favored in this setting because the medialized, deltoid-dependent construct does not rely on cuff integrity and provides reliable fixation in eroded, osteoporotic bone. The rotator cuff in these patients ranges from intact to attenuated to torn, and most published series do not report outcomes by cuff competence so their results cannot be treated as pure cuff-intact data.

However, the available data report favorable outcomes in this population. Lévigne et al., in a multicenter series of 65 primary rTSAs performed for rheumatoid arthritis reported 96% revision-free survivorship at seven years along with improvements in the adjusted Constant score and SSV [33]. Notching occurred in 55% of shoulders and acromial tilt or acromial stress fracture (ASF) in 10%, neither of which significantly affected outcomes [33]. In the only study to directly compare the two procedures in inflammatory arthritis, Garcia et al. found that aTSA produced greater improvements in pain, function, and satisfaction but carried a 7% early revision rate from rotator cuff failure, whereas rTSA showed no significant difference in overall complication rate [34]. Four ASFs occurred in the rTSA group, and excluding those patients minimized the outcome differences between procedures [34]. ATSA carries a high revision risk in this population where rotator cuff failure with proximal humeral head migration occurs in 42 − 55% of rheumatoid patients and produces eccentric glenoid loading that accelerates glenoid loosening [35, 36]. ATSA glenoid loosening is frequent in this population, reaching 40% radiographic loosening at a mean of 92 months and 73% periprosthetic lucency in large rheumatoid series [35, 37]. These findings support rTSA as a favorable option in inflammatory arthritis, particularly when cuff or bone compromise makes anatomic reconstruction difficult, though ASFs and scapular spine stress fractures (SSFs) remain a relevant risk given the poor bone quality in this population.

### Avascular Necrosis

ATSA remains the standard treatment for avascular necrosis (AVN) of the humeral head. McLaughlin et al. compared 67 rTSA and 52 aTSA shoulders treated for AVN from a multicenter database and found significant improvement in all ROM and PROs with both procedures, no differences exceeding the MCID, and low complication rates (3% rTSA, 2% aTSA) [38]. The rTSA cohort was older and included 33% with cuff insufficiency, whereas none of the aTSA patients had significant rotator cuff pathology [38]. This data supports rTSA as a reasonable alternative when other patient-factors may make aTSA less suitable, with comparable pain relief and function.

## Biomechanics of Reverse Total Shoulder Arthroplasty with Intact Rotator Cuff

RTSA was originally designed for the rotator cuff-deficient shoulder. Grammont’s prosthesis used a medialized center-of-rotation to reduce shear forces on the glenoid baseplate and recruit more of the anterior and posterior deltoid for abduction, a constrained articulation with no radius-of-curvature mismatch between the glenosphere and polyethylene liner to prevent superior migration of the humerus under deltoid pull, and a 155**°** neck shaft angle (NSA) to lengthen the deltoid thereby increasing its moment arm [39]. However, these changes shorten the normal rotator cuff muscle-tendon length (due to medialization) and alters its line of pull (due to distalization), reducing its ability to generate IR and ER force [40, 41]. Furthermore, increasing humeral distalization increases the deltoid moment arm but may impair deltoid force generation by causing the muscle to operate outside the optimal portion of its force-length (Blix) curve. When rTSA is performed in the cuff-intact patient, this raises the question of whether modifying implant geometry and surgical technique to maintain more anatomic rotator cuff muscle-tendon lengths and vectors can improve function.

Four primary components of rTSA implant design and surgical technique affect arm and center-of-rotation position and therefore deltoid and rotator cuff function: the humeral NSA, humeral lateralization, glenoid lateralization, and glenosphere position [42, 43]. These parameters affect both the physics of the articulation (moment and lever arms) and the in-vivo function of the muscles (muscle-tendon lengths and the Blix curve). Levin et al. assessed which combinations of these four variables minimally altered native deltoid and rotator cuff muscle-tendon lengths through 0 **−** 90**°** of scaption (FE in the scapular plane) [44]. Their findings differed by shoulder size but generally favored a centrally rather than inferiorly placed 30**−** 36 mm glenosphere, a 135**°** NSA, 5**−** 10 mm of glenoid lateralization, and 0 mm of humeral offset. A computational model reported that a rTSA implanted in a shoulder with a functional rotator cuff, or with an isolated supraspinatus tear, generated more than twice the compressive joint reaction force produced with a massive cuff tear, with comparable shear forces [45]. Continued research is needed to determine how more anatomic positioning of the deltoid and rotator cuff affects function, outcomes, and complications. Importantly, because rTSA recruits distinct muscle activation patterns from the native shoulder with a predominance of scapulothoracic motion, design changes should ultimately be evaluated by clinical outcomes and PROs.

## Implant Design and Surgical Considerations

### Neck-Shaft Angle

The humeral NSA is a key design consideration, affecting humeral lateralization without changing the center of rotation and thereby determining final humeral position and the moment arms of the deltoid and rotator cuff tendons. In an evaluation of 486 implant and shoulder-size combinations, a 135º NSA was the strongest design predictor of restoring anatomic muscle-tendon lengths [44]. A 155º NSA typically shortens the rotator cuff moment arms through medialization of the humerus inherent to the higher NSA and leads to significant alteration in their direction of force generation. Maxwell et al. found that a 135º NSA with an 8 mm lateralized glenosphere produced the longest subscapularis, infraspinatus, and teres minor lengths compared with 145º or 155º configurations [46]. Although computational and biomechanical studies favor a 135º NSA, whether this translates to improvements in ROM, strength, or PROs is unclear. Comparisons of 135° versus 145° NSA have shown lower scapular notching with the 135° stem (30% vs. 53.4%; *P* = 0.028; OR 4.1 with 145° NSA after adjusting for glenoid lateralization and inferiorization) but no difference in Constant-Murley Score, SSV, FE, abduction, ER, and IR at minimum 2-year follow-up [47].

### Lateralization

Lateralization of rTSA can refer to glenoid-sided, humeral-sided, or global lateralization. Lateralization can be achieved through the type of implants used and surgical technique (amount of glenoid reaming, BIO-RSA, stem position, etc.). Glenoid lateralization improves impingement-free ROM in cadaveric and computational studies, though large amounts may add no additional benefit over ‘moderate’ lateralization, with one computational model demonstrating equivalent ROM between 4 and 12 mm glenoid lateralization [48]. Moderate lateralization (5**−** 10 mm) increases joint compressive stability with a small (0.2 **−** 1.8%) increase in acromial cortical bone regions exceeding yield stress when paired with an inlay stem [49]. The increased joint compressive stability of glenoid lateralization may improve overall stability of the prosthesis. Glenoid lateralization increases the deltoid force required to abduct the arm, which may impact ASF and SSF rates [50]. There is also increased shear-force across the bone-implant interface with increasing lateralization. Lateralization of 5**−** 10 mm generally maintains native rotator cuff muscle-tendon lengths without increasing their moment-arms [43, 51]. Humeral lateralization improves rotational ROM, which in cuff-intact patients may reflect increased rotational moment-arms of the rotator cuff.

### Inlay Versus Onlay Humeral Component

Due to the variations in humeral head osteotomy and humeral stem positioning, inlay versus onlay humeral component may not produce consistent biomechanical differences in rTSA. Polyethylene thickness or spacer trays can convert an inlay into an onlay, with many studies reporting conflicting results or no difference in ROM and PROs [52, 53]. From a biomechanical perspective, inlay versus onlay is better captured by the amount of humeral lateralization and distalization related to the NSA, liner thickness, and the level of the humeral head osteotomy [48, 54].

Inlay humeral stems allow preservation of the rotator cuff insertion without significantly increasing humeral offset. When paired with a lateralized glenoid component, either an inlay humeral component or a deeper humeral osteotomy with an onlay component can achieve similar global lateralization [55].

In a biorobotic simulator, rotator cuff forces during scapular-plane abduction were lower with inlay than onlay implants [56]. Onlay stems increased acromial stress by roughly 23–30% compared with inlay configurations, though this has not been shown to consistently affect stress fracture rates [49]. The New Zealand registry reported lower medium-term revision rates with inlay designs (0.665 vs. 1.010 per 100 component-years), despite slightly lower 6-month functional scores and reduced passive FE [57].

### Glenosphere Placement

Biomechanical studies have shown that smaller glenospheres matched to patient shoulder size and placed centrally in the glenoid better restore native rotator cuff muscle-tendon lengths [44]. As with the trade-off between anatomic rotator cuff positioning and preferential deltoid lengthening, inferior glenosphere placement increases the deltoid moment arm, lowers joint reaction forces, and reduces the deltoid force needed for FE, independent of diameter [58]. Inferior placement also reduces notching with little isolated effect on joint stability [59].

### Subscapularis Repair

The decision to repair the subscapularis during rTSA is particularly relevant in the primary GHOA population where most patients do not have severe preexisting subscapularis pathology. Numerous studies have evaluated the impact of subscapularis repair on implant stability, ROM, and PROs. It is important to note that differences in implant center-of-rotation, lateralization, and distalization may affect both the rate and impact of subscapularis healing, and these studies should be interpreted within the context of the implant system used. Among repaired subscapularis tendons, two sonographic studies demonstrated healing rates of only 53% at 3 years after surgery [60, 61], while another study reported complete tendon integrity in just 13% of cases at median 19 months after rTSA [62]. Many systematic reviews and randomized trials have failed to show improvements in PROs with subscapularis repair, though these studies have significant heterogeneity in rTSA indications, implant design, and assessment of healing [63, 64].

Chang et al. evaluated a selective repair protocol in 80 patients undergoing rTSA for primary GHOA with an intact rotator cuff using a lateralized glenoid and inlay humerus design, repairing the subscapularis only when it did not limit intraoperative passive ER [65]. There were no differences in PROs or instability at minimum 1-year follow-up [65]. Repair produced significantly greater IR motion and IR strength, as well as a higher proportion of patients reaching the patient acceptable symptom state for IR [65]. In contrast, no repair produced greater improvement in active ER and a higher proportion reaching substantial clinical benefit for ER [65].

Subscapularis repair outcomes also differ by implant lateralization. In a meta-analysis of 19 articles comparing repair with no repair, Daher et al. found that the reduction in instability with repair was confined to medialized constructs with no difference in the lateralized subgroup—most studies used “lateralized” to refer to glenoid-sided lateralization [66]. They additionally found no difference in IR improvement with subscapularis repair using a lateralized implant [66]. An earlier systematic review similarly found that the instability reduction with repair was limited to medialized implants with the improvement in ASES and Constant scores not exceeding MCID [67]. A lateralized-implant meta-analysis by Corona et al. similarly found higher ASES and Constant scores with repair but no difference in IR, ER, or dislocation, and greater abduction in unrepaired shoulders [68]. Overall, subscapularis repair and healing generally have an equivocal effect on ROM and PROs, with two recent studies showing small and clinically unclear benefits to active IR and IR strength [60–62].

### Cosmesis

Cosmetic appearance is not a reported outcome measure in the rTSA literature, and no study has assessed scar, deltoid contour, or arm-length symmetry as an endpoint in this population. Medialization and distalization of the humerus alters deltoid contour and effective arm position, typically perceived as a loss of shoulder width [54, 69, 70]. Intact rotator cuff shoulders do not show the contour squaring and proximal humeral migration of cuff tear arthropathy, so there is no preoperative cosmetic deformity for rTSA to correct.

## Functional Outcomes and Comparison with Anatomic Total Shoulder Arthroplasty

The central concern in the intact rotator cuff population is whether rTSA can match the functional results of aTSA, and the best comparative evidence comes from large single-implant registries. Friedman et al. matched 370 rTSA and 370 aTSA patients with primary GHOA, an intact rotator cuff, and no prior surgery [6]. At mean 41 month follow-up, both groups achieved good pain relief, high satisfaction, and comparable function, with the only clinically meaningful difference being greater ER in the aTSA group [6]. Humeral radiolucent lines (8%) and revision rates (aTSA = 3.2%; rTSA = 1.4%) were similar between groups, but complications were significantly lower after rTSA (aTSA = 4.9%; rTSA = 2.2%; *P* = 0.045) [6].

Nazzal et al. corroborated these findings, reporting that rTSA performed with posterosuperior cuff preservation for primary GHOA produced outcomes comparable to aTSA except for slightly diminished IR (5.0 vs. 6.5) and ER (38° vs. 44°) [42]. At the population level, a meta-analysis of 14 studies restricted to primary cuff-intact GHOA found that rTSA carried lower complication (OR 0.54) and reoperation rates (OR 0.31) and superior scores for several PROs (Shoulder Pain and Disibility Index, Univeristy of California Los Angeles, and Simple Shoulder Test), while aTSA yielded better ER [71]. For the cuff-intact patient, choosing rTSA over aTSA exchanges a measurable but often clinically acceptable reduction in rotational ROM for lower complication and revision rates (Table 1). These retrospective comparisons are subject to selection bias, since surgeons typically weigh patient demographics and pathology together when choosing between aTSA and rTSA in the cuff-intact patient.

**Table 1:** Recent literature reporting functional outcomes and range of motion for reverse total shoulder arthroplasty (rTSA) in patients with an intact or functional rotator cuff

Study (Year)	Population (*N*, mean age)	Mean Follow-up	Indication	Key Findings
Friedman et al. (2022) [6]	370 rTSA vs. 370 aTSA (matched), mean age 73 years	41 months	Primary GHOA, intact rotator cuff, no prior surgery	Similar PROs and function; pain relief excellent and satisfaction high in both groups; similar ROM except greater ER in aTSA (52.7° vs. 37.9°)
Nazzal et al. (2023) [42]	Rotator cuff-preserving rTSA cohort, mean age 71.1 years	16.2 months	Primary GHOA, intact rotator cuff	rTSA with rotator cuff preservation matched aTSA except for slightly lower IR (5.0 vs. 6.5) and ER (38° vs. 44°)
Daher et al. (2024) [71]	14-study meta-analysis (958 rTSA, 1457 aTSA)	3.4 years	Primary GHOA, intact rotator cuff	rTSA superior UCLA, SST, SPADI; aTSA superior in ER (MD = − 9.0°)
Polisetty et al. (2021) [5]	63 rTSA vs. 252 aTSA, mean age 73 years	38 vs. 54 months	Primary GHOA, intact rotator cuff	Similar outcome improvement in MCIDs, cost measures, patient satisfaction, and FF, with improved rotational motion for aTSA (ER 49.3° vs. 42.0°)
Pettit et al. (2022) [88]	197 rTSA, mean age 71.8 years	28.0 months	Primary GHOA, varied glenoid morphology	98.0% reached MCID and 90.9% substantial clinical benefit for ASES regardless of glenoid deformity; no differences among Walch subtypes in terms of preoperative to postoperative improvement
Polisetty et al. (2023) [22]	101 rTSA vs. 101 aTSA (matched), mean age 71 years	39 months	B2/B3 glenoid, intact rotator cuff	Comparable outcomes; rTSA marginally lower pain (0.5 vs. 1.2, *P* = 0.036), less glenoid radiolucency
Ardebol et al. (2024) [16]	37 rTSA vs. 67 aTSA, mean age 79 years	39.4 months	Primary GHOA, age ≥ 75 years	Similar PROs and clinical improvement after rTSA and aTSA in patients over 75 years
Aleisawi et al. (2025) [30]	36-study meta-analysis (478 rTSA vs. 1349 aTSA), mean age 72 years	4.2 vs. 4.9 years	B2/B3 glenoid, intact rotator cuff	Comparable ASES, Constant, VAS, and ROM improvements
Kim et al. (2022) [89]	Systematic review/6-study meta-analysis (129 rTSA vs. 318 aTSA), pooled mean age 66–78 years	Pooled 24–97 months	Primary GHOA, intact rotator cuff	Comparable SSV, Constant score, SST, and ASES; comparable ROM except increased ER with aTSA (MD = − 10.28°)
Turnbull et al. (2024) [90]	46 bilateral aTSA vs. 9 aTSA/rTSA, mean age 67 vs. 71 years	2 years minimum	Bilateral GHOA, intact rotator cuff	aTSA/rTSA had superior outcome scores, overhead motion, and active abduction compared to patients that underwent aTSA/aTSA; no differences in ER and IR scores or complication rates between groups
Cuff et al. (2023) [9]	93 rTSA vs. 101 aTSA with high-sided reaming), mean age 69 vs. 66 years	7.8 vs. 8.3 years	Eccentric (B/C) wear, intact rotator cuff	aTSA better outcome scores and ROM with a similar patient satisfaction at 2 years; rTSA superior outcome scores and patient satisfaction with lower revision rates at 7-years (satisfaction 95% vs. 82%)
Menendez et al. (2022) [91]	1758 rTSA vs. 2693 aTSA from Surgical Outcomes System global registry, mean age 70.9 years	Up to 5 years	Primary GHOA, intact rotator cuff	Increased adjusted ASES score with aTSA; comparable adjusted SANE and VAS score
Wright et al. (2020) [19]	33 rTSA vs. 102 aTSA, mean age 78 vs. 77 years	57 vs. 80 months	Primary GHOA, image confirmed intact rotator cuff, < 90° FF, ≥70 years	Similar PROs, pain, satisfaction, ROM; 100% rTSA vs. 98% aTSA rated FF as full or nearly full (> 135°) (*P* = 0.516)
Trammell et al. (2023) [18]	163 rTSA vs. 344 aTSA, mean age 71.7 vs. 65.0 years	3.4 vs. 5.9 years	Primary GHOA, intact rotator cuff, ≤105° vs. >105° passive FF	No difference in postoperative ROM for stiff and non-stiff rTSA; stiff rTSA achieved significantly greater FF and abduction, pain, and PROs than stiff aTSA
Karimi et al. (2024) [20]	57 rTSA vs. 59 aTSA, restricted FF, mean age 72.0 vs. 65.4 years	20.1 vs. 17.7 months	Primary GHOA, restricted FF < 120° vs. <90°	Similar 2-year pain, PROs and ROM except lower IR in rTSA; faster ROM recovery with rTSA
Hao et al. (2023) [92]	87 stiff rTSA vs. 263 non-stiff rTSA, mean age 72.7 vs. 72.8 years	41.8 vs. 66.2 months	Primary GHOA, subgroups of passive ER stiffness ≤0°	Comparable PROs; stiff patients had higher postoperative IR and ER when treated with aTSA

GHOA = glenohumeral osteoarthritis; aTSA = anatomic total shoulder arthroplasty; rTSA = reverse total shoulder arthroplasty; ASES = American Shoulder and Elbow Surgeons; ER = external rotation; FF = forward flexion; IR = internal rotation; MD = mean difference; MCID = minimal clinically important difference; PROs = Patient Reported Outcomes; SCB = substantial clinical benefit; SPADI = Shoulder Pain and Disability Index; SST = Simple Shoulder Test; UCLA = University of California Los Angeles shoulder score; SSV = Subjective Shoulder Value

## Complications

### Scapular Notching

With modern lateralized, lower-inclination designs, scapular notching occurs in roughly 5–11% of cuff-intact rTSA patients, below the 50 **−** 86% reported with original Grammont-style implants [8,17,72] and the 10 **−** 22% seen with modern implants with cuff-deficient populations [72, 73]. Notching is design-dependent where a 135° NSA with glenoid lateralization ≥ 6 mm reduces rates to 5–11%, and inferior glenosphere placement with adequate overhang is additionally independently protective [74, 75]. An intact rotator cuff lowers overall complication rates (OR 0.57, *P* < 0.001) but does not eliminate notching [8]. When notching occurs, Simovitch et al. found patients had worse ASES and Constant scores, abduction, FE, and strength at minimum 5-year follow-up, with more complications and revisions [76]. Inferior glenosphere position, lateralization ≥ 6 mm, and avoiding superior tilt reduce notching regardless of rotator cuff status [75, 77].

### Acromial and Scapular Spine Stress Fractures

Acromial (ASF) and scapular spine (SSF) stress fractures occur in 2.1 **−** 2.8% of intact rotator cuff rTSA patients, roughly 2.5-fold lower than the 5.2 **−** 7.4% in cuff-deficient populations [78, 79]. Daher et al. quantified this protective effect as an OR of 0.22 (*P* = 0.003) [8]. In a 2023 study by the ASES Complications of RSA Research Group, cuff-deficient patients were at elevated risk for ASF an SSF if female, had osteoporosis, or had inflammatory arthritis, whereas in the cuff-intact cohort the only risk factor was inflammatory arthritis [78]. How implant design affects stress fracture risk remains debated. Medialized glenoid configurations have demonstrated to lower rates of ASF and SSF, while greater global lateralization increases fracture risk (OR 1.06 per mm, *P* = 0.025) [80]. Humeral-sided lateralization has been shown to be protective (OR 0.74, *P* = 0.031) [80]. However, Pak et al. found that up to 8 mm of glenoid-sided lateralization did not increase the risk when combined with a 135° inlay humeral component (*P* = 0.89), with fracture rates ranging from 4.4 to 7.4% [81]. Eisenberg et al. reduced fracture rates from 9.2% to 2.3% by selectively medializing the glenoid in high-risk patients (massive cuff tear, cuff tear arthropathy, inflammatory arthritis, proximal humerus sequelae) [82].

### Component Loosening, Instability, and Revision Rates

Glenoid baseplate loosening is the leading cause of revision after rTSA in cuff-intact GHOA, accounting for 40% of revisions, compared with aTSA where rotator cuff tear accounts for 54% [83]. Walch B2 and B3 glenoids, common in cuff-intact GHOA, are themselves predictors of baseplate loosening, as are augmented baseplates and expanded lateralized glenospheres [25]. In a cuff-intact GHOA series treated with eccentric reaming and no bone grafting, the cumulative incidence of baseplate failure requiring revision was 2.4% at 2 years and 5.2% at 5 years [84]. In a matched multi-institutional series of rTSA for cuff-intact GHOA, the revision rate was 1.4% at a mean of 41 months [6]. At this time, no studies yet evaluate long-term survival over 10 years for rTSA in cuff-intact patients. An updated New Zealand registry of 6,862 rTSA reported 10-year revision-free survival for GHOA of 96.1%, versus 93.7% for cuff tear arthropathy and 92.8% for rheumatoid arthritis [85].

Dislocation is uncommon in cuff-intact patients. In the ASES multicenter analysis of 6,621 rTSAs, the overall dislocation rate was 2.1%, and primary GHOA patients with an intact rotator cuff had a lower rate of dislocation than patients with other diagnoses (0.8% vs. 2.5%, *P* < 0.001) [78]. Cuff tear arthropathy or massive tear is an independent risk factor for dislocation relative to intact rotator cuff diagnoses (OR 2.64) [78]. The meta-analysis by Daher et al. similarly found lower instability in cuff-intact patients (OR 0.60, *P* = 0.01) [8]. When dislocation occurs, closed reduction succeeds in only 31% of cases and 66.7% ultimately require revision [86].

In a 2025 meta-analysis restricted to cuff-intact GHOA, rTSA demonstrated a significantly lower revision rate than aTSA (OR 0.46, *P* = 0.0003), with comparable outcomes and ROM except for ER (Table 2) [87]. Pooled revision rates were 1.7% for rTSA versus 3.9% for aTSA [87]. Daher et al. also found a lower revision rate in cuff-intact shoulders than in cuff-deficient shoulders (OR 0.53, *P* < 0.001) [8]. In patients ≥ 70 years, Orvets et al. found no difference in all-cause revision between rTSA and aTSA [83].

**Table 2:** Selected studies reporting complication and revision outcomes of rTSA in rotator cuff intact osteoarthritis and in inflammatory arthritis

Study (Year)	Population (*N*, mean age)	Mean Follow-up	Indication / Comparator	Key Findings
Overall Complication / Revision Rates (GHOA with Intact Rotator Cuff)				
Friedman et al. (2022) [6]	370 rTSA vs. 370 aTSA (matched), mean age 73 years	41 months	Primary GHOA, intact rotator cuff, no prior surgery	Complications lower with rTSA (2.2% vs. 4.9% aTSA, *P* = 0.045); scapular notching in 9.9% of rTSA and glenoid radiolucent lines in 16.8% of aTSA; revisions similar (rTSA 1.4% vs. aTSA 3.2%, *P* = 0.086)
Nazzal et al. (2023) [42]	24 rotator cuff-preserving rTSA vs. 93 aTSA, mean age 71.1 vs. 66.0 years	16.2 vs. 17.7 months	Primary GHOA, intact rotator cuff	Complications lower with rTSA (4.2%; PJI) vs. aTSA (overall 12.9%)
Daher et al. (2024) [71]	14-study meta-analysis (958 rTSA, 1457 aTSA)	3.4 years	Primary GHOA, intact rotator cuff	rTSA carried lower complication (OR 0.54; 3.5% vs. 8.2%) and reoperation rates (OR 0.31) than aTSA; most common complications of glenoid or periprosthetic fracture, or PJI (0.4% incidence each)
Polisetty et al. (2021) [5]	63 rTSA vs. 252 aTSA, mean age 73 years	Radiographs at 30.1 vs. 36.0 months	Primary GHOA, intact rotator cuff	No areas of radiolucencies were identified in rTSA vs. 53 (21%, *P* < 0.001) in aTSA in at least 1 area around polyethylene glenoid component with 8 (3%) with gross loosening; No revisions were performed in the rTSA cohort
Pettit et al. (2022) [88]	197 rTSA, mean age 71.8 years	28.0 months	Primary GHOA, varied glenoid morphology	1 (0.50%) revision (B3 glenoid) for baseplate failure; 8 (4.06%) orthopedic complications; 1 (0.51%) transient radial nerve palsy (C); 1 (0.51%) persistent brachial plexopathy (A1); 2 (1.02%) ASFs (A1, B2); 2 (1.02%) baseplate failures (B2, B3); 1 (0.51%) intraoperative greater tuberosity fracture (A1)
Polisetty et al. (2023) [22]	101 rTSA vs. 101 aTSA (matched), mean age 71 years	39 months	B2/B3 glenoid, intact rotator cuff	4 (4.0%) rTSA complications (traumatic acromial fracture, transient neuropathy, transient cubital tunnel syndrome, and revision for traumatic glenoid loosening); 3 (3.0%) aTSA complications
Reahl et al. (2021) [26]	B2 glenoid meta-analysis (78 rTSA vs. 414 aTSA), mean age 72.8 vs. 63.7 years	44.8 vs. 54.1 months	Walch B2 glenoid, no significant rotator cuff pathology	Complications 9% (aTSA) vs. 6% (rTSA); most common complication of nerve palsy (4%)
Aleisawi et al. (2025) [30]	36-study meta-analysis (478 rTSA vs. 1349 aTSA), mean age 72 years	4.2 vs. 4.9 years	B2/B3 glenoid, intact rotator cuff	Complication 4.4% (rTSA) vs. 7.8% (aTSA) ; revision 1.6% (rTSA) vs. 5.2% (aTSA)
Cuff et al. (2023) [9]	93 rTSA vs. 101 aTSA with high-sided reaming), mean age 69 vs. 66 years	7.8 vs. 8.3 years	Eccentric (B/C) wear, intact rotator cuff	2 (2%) rTSA revisions (dislocation, PJI) vs. 10 (8%) aTSA at 2 years; rTSA with 2 (2%) ASFs and 1 (1%) axillary nerve palsy
Trammell et al. (2023) [18]	99 stiff rTSA vs. 125 non-stiff rTSA, mean age 71.7 vs. 71.4 years	3.4 vs. 3.6 years	Primary GHOA, intact rotator cuff, ≤105° vs. >105° passive FF	6 (6.9%) stiff rTSA complications (periprosthetic fracture, intraoperative humeral fracture; 2% each) vs. 12 (9.6%) non-stiff rTSA (*P* = 0.46) (glenoid loosening; 4%)
Hao et al. (2023) [92]	87 stiff rTSA vs. 263 non-stiff rTSA, mean age 72.7 vs. 72.8 years	41.8 vs. 42.6 months	Primary GHOA, subgroups of passive ER stiffness ≤0°	2 (2.3%) stiff rTSA complications vs. 6 (2.3%) non-stiff rTSA; 1 (1.1%) stiff rTSA revisions vs. 3 (1.1%) non-stiff rTSA

GHOA = glenohumeral osteoarthritis; aTSA = anatomic total shoulder arthroplasty; rTSA = reverse total shoulder arthroplasty; PJI = prosthetic joint infection; ASF = acromial stress fracture

In addition to lower risk of revisions and instability, the large meta-analysis by Daher et al. also found that an intact rotator cuff with rTSA was associated lower overall complications (OR 0.57), ASFs (OR 0.22), and infection (OR 0.43) (all *P* ≤ 0.002) compared with cuff-deficient shoulders [8].

## Conclusions

RTSA is a safe, reliable, and durable option for primary GHOA with an intact rotator cuff. In matched cohorts and meta-analyses restricted to cuff-intact GHOA, rTSA and aTSA produce comparable pain relief, PROs, satisfaction, and overhead elevation. ATSA preserves more ER and IR, but the difference often falls below clinically important thresholds and narrows over time. ATSA and rTSA fail by different mechanisms. Rotator cuff dysfunction and glenoid polyethylene loosening drive late aTSA failure. Baseplate loosening is the most common cause of rTSA revision in the cuff-intact population primarily because rates of instability, acromial and scapular spine stress fracture, and infection are significantly lower than in rotator cuff tear arthropathy patients. No long-term survivorship outcomes greater than 10 years have been published for rTSA with an intact rotator cuff, but registry survivorship of rTSA for GHOA is as high as 96.1% at 10 years. Implant design may be most successful when a balance is achieved between implant configurations that optimize the moment-arms and muscle-tendon lengths of both the deltoid and the rotator cuff. Moderate amounts of glenoid and humeral lateralization are safe, optimizes function, and minimizes complications in cuff-intact patients. Repair of a subscapularis that does not restrict passive external rotation may improve outcomes when there is successful tendon healing. Ultimately, further comparative studies are still needed to determine when rTSA should be chosen over aTSA, and which implant configurations will yield optimal patient outcomes.

## Key References

- Friedman RJ, Schoch BS, Eichinger JK, Neel GB, Boettcher ML, Flurin PH, et al. Comparison of Reverse and Anatomic Total Shoulder Arthroplasty in Patients With an Intact Rotator Cuff and No Previous Surgery. J Am Acad Orthop Surg. 2022;30:941–8. https://doi.org/10.5435/JAAOS-D-22-00014 PubMed PMID: 36,135,928.
  - ○ This was a propensity-matched study drawn from an international multi-institutional registry comprising 740 patients (370 aTSA vs. 370 rTSA; mean age 73 years) with primary GHOA, an intact rotator cuff, and no prior surgery, using a single implant platform with a minimum 2-year follow-up (mean 41 months). Both groups achieved excellent pain relief, high satisfaction, and comparable function, with the only clinically meaningful difference being greater ER in the aTSA group. Humeral radiolucent lines (8%) and revision rates (aTSA 3.2% vs. rTSA 1.4%) were similar, but complications were significantly lower after rTSA (2.2% vs. 4.9%; *P* = 0.045). As one of the largest matched comparisons restricted specifically to the cuff-intact population, this study establishes that reverse and anatomic arthroplasty deliver equivalent early clinical outcomes in this group, with reverse arthroplasty conferring a lower complication burden at the expense of a measurable but often subclinical reduction in rotation.
- Daher M, Ashkar I, Parmar T, Kane L, Satalich JR, Klifto CS, et al. The Impact of an Intact Rotator Cuff on the Outcomes of Reverse Shoulder Arthroplasty: A Meta-Analysis of 20,924 Patients. J Shoulder Elbow Surg. 2026;S1058-2746(26)00165-5.https://doi.org/10.1016/j.jse.2026.03.023 PubMed PMID: 41,966,468.
  - ○ This was a systematic review and meta-analysis of 12 studies comprising 20,924 patients that compared outcomes of rTSA performed for GHOA with an intact rotator cuff (4,542 patients) versus cuff-deficient shoulders (16,382 patients). Cuff-intact patients had significantly lower rates of revision (OR 0.53), overall complications (OR 0.57), acromial stress fracture (OR 0.22), infection (OR 0.43), and instability (OR 0.60), along with greater improvement in ASES scores and ER, though several differences did not exceed thresholds for clinical importance. These findings quantify the protective effect of an intact rotator cuff across the major complication categories and provide the strongest pooled evidence that the favorable complication profile of rTSA in primary GHOA is driven substantially by rotator cuff competence rather than the implant alone.
- Cuff DJ, Simon P, Patel JS, Munassi SD. Anatomic shoulder arthroplasty with high side reaming versus reverse shoulder arthroplasty for eccentric glenoid wear patterns with an intact rotator cuff: comparing early versus midterm outcomes with minimum 7 years of follow-up. J Shoulder Elbow Surg. 2023;32:972–9. doi:https://doi.org/10.1016/j.jse.2022.10.017 PubMed PMID: 36,400,340.
  - ○ This was a retrospective comparison of 194 shoulders with eccentric (Walch B/C) glenoid wear and an intact rotator cuff treated with aTSA and high-side reaming (aTSA + HSR, *n* = 101) or rTSA (rTSA, *n* = 93), followed to a minimum of 7 years (mean 8.3 vs. 7.8 years). At 2 years, aTSA + HSR produced superior ASES scores, FE (162° vs. 151°), ER (47° vs. 42°), and IR (80% vs. 55% full IR; all *P* < 0.001), but these advantages diminished over time. At final follow-up, FE had converged (152° vs. 149°), the rTSA cohort had higher ASES scores (80 vs. 77) and patient satisfaction (95% vs. 82%), and a lower revision rate (2% vs. 8%). These findings provide some of the longest comparative follow-up in the cuff-intact population and demonstrate that the early rotational advantage of aTSA diminishes by midterm, while rTSA offers more durable outcomes and fewer revisions.
- Trammell AP, Hao KA, Hones KM, Wright JO, Wright TW, Vasilopoulos T, et al. Clinical outcomes of anatomical versus reverse total shoulder arthroplasty in patients with primary osteoarthritis, an intact rotator cuff, and limited forward elevation. Bone Jt J. 2023;105-B:1303–13. doi:https://doi.org/10.1302/0301-620X.105B12.BJJ-2023-0496.R2 PubMed PMID: 38,037,676.
  - ○ This was a retrospective cohort study of 507 shoulders (344 aTSA vs. 163 rTSA) with primary GHOA and an intact rotator cuff, in which preoperative stiffness was defined as passive forward elevation ≤ 105° and three cohorts were matched 1:1 by age, sex, and follow-up: stiff versus non-stiff aTSAs, stiff versus non-stiff rTSAs, and stiff aTSAs versus stiff rTSAs. Whereas stiff aTSAs showed poorer postoperative passive forward elevation and ER than non-stiff aTSAs, stiff rTSAs performed comparably to their non-stiff counterparts. In the direct comparison, stiff rTSAs achieved significantly greater postoperative active and passive forward elevation and active abduction than stiff aTSAs, with more favorable SPADI, SST, ASES, UCLA, and Constant scores, a similar complication rate, but a significantly lower rate of revision surgery (*P* = 0.007). These findings identify limited preoperative forward elevation as a scenario in which rTSA yields superior motion and outcome scores with fewer revisions, supporting its selection over aTSA in patients whose stiffness may reflect a functionally impaired rotator cuff.

## Data Availability

No datasets were generated or analysed during the current study.
